# Prognostic impact of hepatitis B virus infection in patients with primary cervical cancer

**DOI:** 10.1002/cam4.4358

**Published:** 2021-10-21

**Authors:** Xiaoyan Feng, Huaiwu Lu, Yuan Wei, Meimei Guan, Junyi Wang, Changhao Liu, Tianran Shen, Qingsong Chen, Qunxian Rao

**Affiliations:** ^1^ Guangdong Provincial Engineering Research Center of Public Health Detection and Assessment Guangdong Pharmaceutical University Guangzhou China; ^2^ School of Public Health Guangdong Pharmaceutical University Guangzhou China; ^3^ Department of Gynaecological Oncology Sun Yat‐Sen Memorial Hospital Guangzhou China

**Keywords:** cervical cancer, hepatitis B virus (HBV), human papillomavirus (HPV), prognosis, surgery

## Abstract

**Background:**

Hepatitis B virus (HBV) infection has been associated with an increased risk of a few malignancies. However, the prognostic impact of HBV infection remains unclear in cervical cancer.

**Objective:**

To explore the association between HBV infection and survival outcomes of patients with primary cervical cancer, using overall survival (OS) and disease‐free survival (DFS) as primary endpoints.

**Methods:**

This analysis was performed retrospectively with newly diagnosed cervical cancer patients admitted to the Department of Gynecologic Oncology at the Sun Yat‐sen Memorial Hospital of Sun Yat‐sen University from June 2013 to October 2019, who were enrolled and followed up. The Kaplan–Meier method and Cox proportional hazard analysis were used to examine the performance of HBV infection in predicting OS and DFS.

**Results:**

Patients were followed up for a median of 37.17 months (95% CI, 34.69–39.65). Among the 695 patients, 87 (12.5%) were serologically positive for hepatitis B surface antigen (HBsAg), and 276 (39.7%) had a prior history of HBV infection. There was no significant difference between HBsAg‐positive group and HBsAg‐negative patients concerning OS or DFS. Multivariate analysis showed prior HBV infection was an independent favorable prognosticator for OS (HR, 0.335; 95% CI, 0.153–0.0.734; *p* = 0.006) and DFS (HR, 0.398; 95% CI, 0.208–0.691; *p* = 0.002).

**Conclusion:**

We provide the first clinical evidence that suggests prior HBV infection as an independent favorable prognostic factor for patients with primary cervical cancer.

## Graphical abstract

Our study was based on a retrospective cohort study, to explore the association between HBV infection and survival outcomes of patients with primary cervical cancer, using overall survival (OS) and disease‐free survival (DFS) as main endpoints. In this study, we find HBsAg has no significance for the prognosis of cervical cancer, and provide the first clinical evidence that previous hepatitis B infection with cervical cancer as an positive effect on the prognosis as an independent favorable prognostic factor for patients with primary cervical cancer.

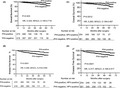

**LAY SUMMARY:**

This study used information from a retrospective cohort of women with cervical cancer to explore the association between HBV infection and survival outcomes of patients with primary cervical cancer.

The results show that HBsAg has no significance for the prognosis of cervical cancer, but prior hepatitis B virus infection with cervical cancer has a positive effect on the prognosis. This findings may help to identify patients for trials of new treatment inventions and studies of potential biological mechanisms.

## 1 INTRODUCTION

Cervical cancer (CC) is a malignant tumor frequently occurring in women, with an estimated 570,000 new cases around the world in 2018.^1^ In particular, approximately 130,000 of these cases are from China, accounting for over one‐fifth of the global tally. Risk factors for cervical cancer include persistent human papillomavirus (HPV) infection, chlamydia trachomatis infection, HIV infection, smoking, and oral use of contraceptives.^2^, ^3^ Among these factors, chronic infection with high‐risk HPV subtypes or with HIV has been suggested to induce carcinogenesis through impairing the host immune response, leading to an immunodeficient state that facilitates tumor initiation and growth.^4^

In addition to HPV and HIV, other factors may also lead to greater risk of the development of CC. One such candidate is infection with hepatitis B virus (HBV). In addition to cancer, HBV infection represents another major threat to public health, with an especially high incidence rate in the East Asian population.^5^ Despite great efforts to control HBV in China over the past 20 years, the carrier rate of hepatitis B surface antigen (HBsAg) among Chinese adults remains high, ranging from 6% to 9.5%.^6^ Aside from hepatitis and liver cancer, more and more evidence indicates that HBV infection is associated with increased susceptibility of a host of extrahepatic malignancies,^7^ including non‐Hodgkin lymphoma,^8^ pancreatic cancer,^9^ gastric cancer,^10^ nasopharyngeal cancer,^11^ and ovarian cancer.^12^ In particular, a hospital registry‐based, the case–control study suggested a significant association between HBV infection and CC.^13^ However, characterization of this association, such as the clinicopathological characteristics associated with HBV‐positive CC patients and the prognostic relevance of HBV infection, awaits further research. In this study, we investigated the value of HBV, including active and previous infection, in predicting the survival outcomes in primary cervical cancer.

## 2 MATERIALS AND METHODS

### 2.1 Study design and patients

This analysis enrolled patients with newly diagnosed cervical cancer admitted to the Department of Gynecologic Oncology at the Sun Yat‐sen Memorial Hospital of Sun Yat‐sen University from June 2013 to October 2019. Inclusion criteria were as follows: the patient was (1) aged ≥18 years old; (2) had histopathologically confirmed primary cervical cancer; (3) received radical surgery for cervical cancer; (4) had hepatitis B status confirmed by HBsAg testing; and (5) had complete medical records. The patient was excluded from this study if one of the following exclusion criteria applied: (1) the patient had a benign cervical tumor; (2) the patient suffered from mental disorders; (3) there were other malignancies; and (4) the patient was pregnant or lactating. The study was conducted by the ethical standards laid down in the Declaration of Helsinki. All participants have signed informed consent, and institutional Review Board approval was obtained from the Medical Ethics Board at the Sun Yat‐sen Memorial Hospital.

Clinicopathologic features documented at baseline included age, risk factors, previous history of HBV infection, number, size, stage, and grade of the cervical lesion(s), histological subtype, HPV genotyping, HBV serum markers, as well as the date and procedure of the surgery. All enrolled patients were followed up via telephone or outpatient service every 3 months for the first year after surgery, every 4–6 months after 2 years, and every year from year 5. Overall survival (OS) and disease‐free survival (DFS) were the endpoints of our study. Routine examinations at each visit included gynecological examination, thin prep cytologic test, HPV test, gynecological B‐ultrasound, and other examinations to search for signs of distant metastasis.

### 2.2 Serological tests for infection with HBV, HPV, HIV, and other hepatitis viruses

Baseline serological test results for HBV infection were retrieved from the medical record system. Serum levels of five HBV markers (HBsAg, HBsAb, HBeAg, HBeAb, and HBcAb) were determined using enzyme‐linked immunosorbent assay (ELISA) following standard procedures. A patient was considered HBV‐positive if her blood tested positive for HBV surface antigen (HBsAg), and PHI‐positive if her results were HBsAg‐negative/HBcAb‐positive or HBsAb‐positive/HBcAb‐positive. HPV tests consisted of detection of the viral DNA and RNA (mRNA E6/E7) via nucleic acid hybridization method and polymerase chain reaction per standard testing procedures. The blood samples were also tested for antibodies against human immunodeficiency virus (HIV), hepatitis A/C/D/E virus, and hepatitis D virus antigens using ELISA.

### 2.3 Cervical biopsy

Cervical biopsy was conducted following standard procedures. Briefly, after wiping the cervical secretions with a cotton swab, it needs to apply acetic acid to the cervix and perform a visual inspection to detect acetowhite areas, which were subsequently examined with colposcopy. Biopsies were taken from suspected lesions, fixed, and subjected to downstream processing for pathological review.

### 2.4 Statistical analysis

Overall survival (OS) was defined as the time between the date of diagnosis and the date of death from any cause. Disease‐free survival (DFS) was defined as the time between the date of diagnosis and the date of disease recurrence/metastasis or death from any cause. Metastasis is defined as cervical cancer cells spread directly from the primary site to adjacent tissues or organs or reach other sites through lymphatic fluid, blood et al. to continue to grow. Recurrence refers to the recurrence of cancer after radical treatment. Cancer appears in the original treatment area and its pathological type is the same as that of original cancer. The *χ^2^* test and multivariate Cox regression were used to evaluate the impact of distinct variables on DFS and OS. The Kaplan–Meier survival plots were used to visualize the DFS and OS between patient groups. All *p*‐values were two‐sided, and *p* > 0.05 was considered statistically significant. Statistical analyses were performed with SPSS software (version 25.0; SPSS Inc.) and the Kaplan–Meier survival plots were performed with GraphPad Prism (version 8.0.2 (263)).

## 3 RESULTS

A total of 695 patients with primary CC were screened in this study, and the demographic and clinicopathologic characteristics of the 695 patients with cervical cancer are shown in Table 1. Hepatitis B surface antigen (HBsAg) was detected in 87 (12.5%) patients. The M (p25–p75) age of the HBsAg‐positive patients was 45 (38–54), and 48 (41–55) for HBsAg‐negative ones. There was no statistically significant age difference between the two groups. In terms of educational background, most patients received secondary education. Approximately 78% of patients had squamous carcinoma, about 94.5% of CC patients had less than or equal to stage IIB. Slightly more patients underwent surgery plus radiotherapy. Consistent with the prominence of HPV infection in CC, 637 (91.7%) patients tested positive for the HPV virus. Notably, for all five abovementioned features, HBsAg positivity appeared highly comparable when patients were stratified by either feature, suggesting independence of HBsAg from these factors.

**TABLE 1:** Demographic and clinicopathologic characteristics of patients analyzed in this study

Characteristics	HBsAg‐positive (*N* = 87) No. (%)	HBsAg‐negative (*N* = 608) No. (%)	*χ* ^2^ *p‐value*
Age, years *M_(P25–P75)_*	45 (38–54)	48 (41–55)	0.283
Educational background			
Primary school and below	27 (31.0)	197 (32.4)	0.249
Middle school/high school	43 (49.4)	332 (54.6)	
College and above	17 (19.5)	79 (13.0)	
histology			
Squamous carcinoma	65 (74.7)	477 (78.5)	0.270
Adenocarcinoma	17 (19.5)	82 (13.5)	
Others	5 (5.7)	49 (8.1)	
FIGO stages			
≤ⅡB	81 (93.1)	576 (94.7)	0.459
>ⅡB	6 (6.9)	32 (5.3)	
Treatment			
Surgery	37 (42.5)	308 (50.7)	0.156
Surgery plus radiotherapy	50 (57.5)	300 (49.3)	
HPV status			
Positive	79 (90.8)	558 (91.8)	0.759
Negative	8 (9.2)	50 (8.2)	

Abbreviation: HPV, human papilloma virus infection.

### 3.1 Prognostic value of HBV infection status in primary CC patients

During the follow‐up period up to 2 September 2020, 43 (6.2%) patients had died and 29 (4.17%) patients had developed metastases. The overall mortality rate of HBsAg‐positive patients was 4.6%, which was numerically but not significantly lower than that of patients with negative surface antigen (6.4%). Aside from HBsAg status, which indicated whether the patient was currently infectious, the relevance of prior HBV infection (PHI) was also investigated. Retrieval of relevant information from the patients’ medical records showed a considerably higher proportion of patients with a history of HBV infection, accounting for 276 (39.7%) of the entire cohort (Table 2). The overall mortality rate was 4.5% in patients with PHI and significantly lower (*χ* ^2^ *p* = 0.003) than that (8.4%) in patients with no known PHI.

**TABLE 2:** The influence of HBV status on the survival of patients with primary cervical cancer

Characteristics	Dead (*N* = 43) No. (%)	Alive (*N* = 652) No. (%)	*χ* ^2^ *p*‐value
HBsAg			
Positive	4 (9.3)	83 (12.7)	0.511
Negative	39 (90.7)	569 (87.3)	
Prior HBV infection^a^			
Positive	8 (18.6)	268 (41.1)	0.003
Negative	35 (81.4)	384 (58.9)	
HBsAg ±HPV infection			
HBsAg‐positive and HPV‐positive	4 (9.3)	75 (11.5)	0.885
HBsAg‐negative and HPV‐positive	39 (90.7)	569 (87.3)	
Others	0 (0.0)	8 (1.2)	
PHI ±HPV infection^b^			0.003
PHI‐positive, HPV‐positive	6 (14.0)	249 (38.2)	
PHI‐negative, HPV‐positive	33 (76.7)	349 (53.5)	
Others	4 (9.3)	54 (8.3)	

Note

Bold values are statistically significant (*p* < 0.05).

Abbreviations: HBsAg, hepatitis B surface antigen; HBcAb, anti‐hepatitis B core antigen; anti‐hepatitis B core antigen; HBsAb, anti‐hepatitis B surface antigen; PHI, prior HBV infection.

^a^ Prior HBV infection was defined as patients with HBV testing profiles HbcAb‐positive and HBsAg‐negative or HbcAb‐positive and HBsAb‐positive.

^b^ Included patients with prior hepatitis B virus infection or human papillomavirus infection.

Next, we examined the prognostic effect of active or prior HBV infection, with or without considering concurrent HPV infection. As detailed in Table 2, among HPV‐positive patients, there were 79 (11.4%) with detected HBsAg. The overall mortality rate of this HPV‐positive, HBsAg‐positive subgroup was 5.1%. In the 255 (36.7%) HPV‐positive, HBsAg‐negative patients, the mortality rate was at a lower 2.4%, although there was no statistically significant difference in the mortality rates between these two patient subgroups. In contrast, a history of HBV infection was associated with reduced mortality in HPV‐positive patients (*χ* ^2^ *p* = 0.003; Table 2).

In addition to overall mortality, the prognostic relevance of current or prior HBV infection was further evaluated using multivariate Cox proportional hazards regression. Univariate Cox analysis was first performed on individual factors to screen for those more likely to be an independent prognosticator, including age, treatment (surgery vs. surgery plus radiotherapy), degree of education (primary school vs. other), histologic subtype (squamous cell carcinomas vs. other), FIGO stages (≤ⅡB vs. >ⅡB), HBsAg status, and prior HBV infection status. All variables with a *p*‐value of no greater than 0.1 and HPV and HBsAg may have clinical value in the univariate analysis were included in the subsequent multivariate Cox regression. As shown in Table 3, patients with PHI had significantly better OS (HR, 0.335; 95% CI, 0.153–0.0.734; *p* = 0.006) and DFS (HR, 0.398; 95% CI, 0.208–0.691; *p* = 0.002) prognosis than those without. Significantly shorter OS (HR, 2.528; 95% CI, 1.270–5.033; *p* = 0.008) and DFS (HR, 2.257; 95% CI, 1.342–3.798; *p* = 0.002) were also observed in patients treated with surgery plus radiotherapy, compared with those who underwent surgery alone, which may have resulted from the more advanced disease status in the patients selected for radiotherapy. No significant risk of death or recurrence was observed for the remaining covariates in the model, including age, HBsAg status, and composite status of PHI and HPV infection.

**TABLE 3:** Prognostic effects indicated by multivariate Cox analysis with selected factors

Variable	Overall survival			Disease‐free survival		
	Hazards ratio	95% CI	*p*	Hazards ratio	95% CI	*p*
Age, years	1.019	0.986–1.052	0.261	1.028	1.003–1.054	0.026
Prior HBV infection						
PBI‐negative		1			1	
PBI‐positive	0.335	0.153–0.0.734	0.006	0.379	0.208–0.691	0.002
Treatment						
Surgery		1			1	
Surgery plus radiotherapy	2.528	1.270–5.033	0.008	2.257	1.342–3.798	0.002
FIGO stages						
≤ⅡB		1			1	
>ⅡB	2.678	0.130–1.087	0.070	1.758	0.695–4.442	0.233
HBsAg						
HBsAg‐negative		1			1	
HBsAg‐positive	0.480	0.167–1.376	0.172	0.978	0.423–2.263	0.958
HPV						
HPV‐negative		1			1	
HPV‐positive	0.863	0.307–2.423	0.779	0.841	0.442–1.675	0.622

Note

PHI, prior hepatitis B virus (HBV) infection; 95% CI, 95% confidence interval bold values are statistically significant (*p* < 0.05).

### 3.2 Subgroup analysis

**FIGURE 1:**
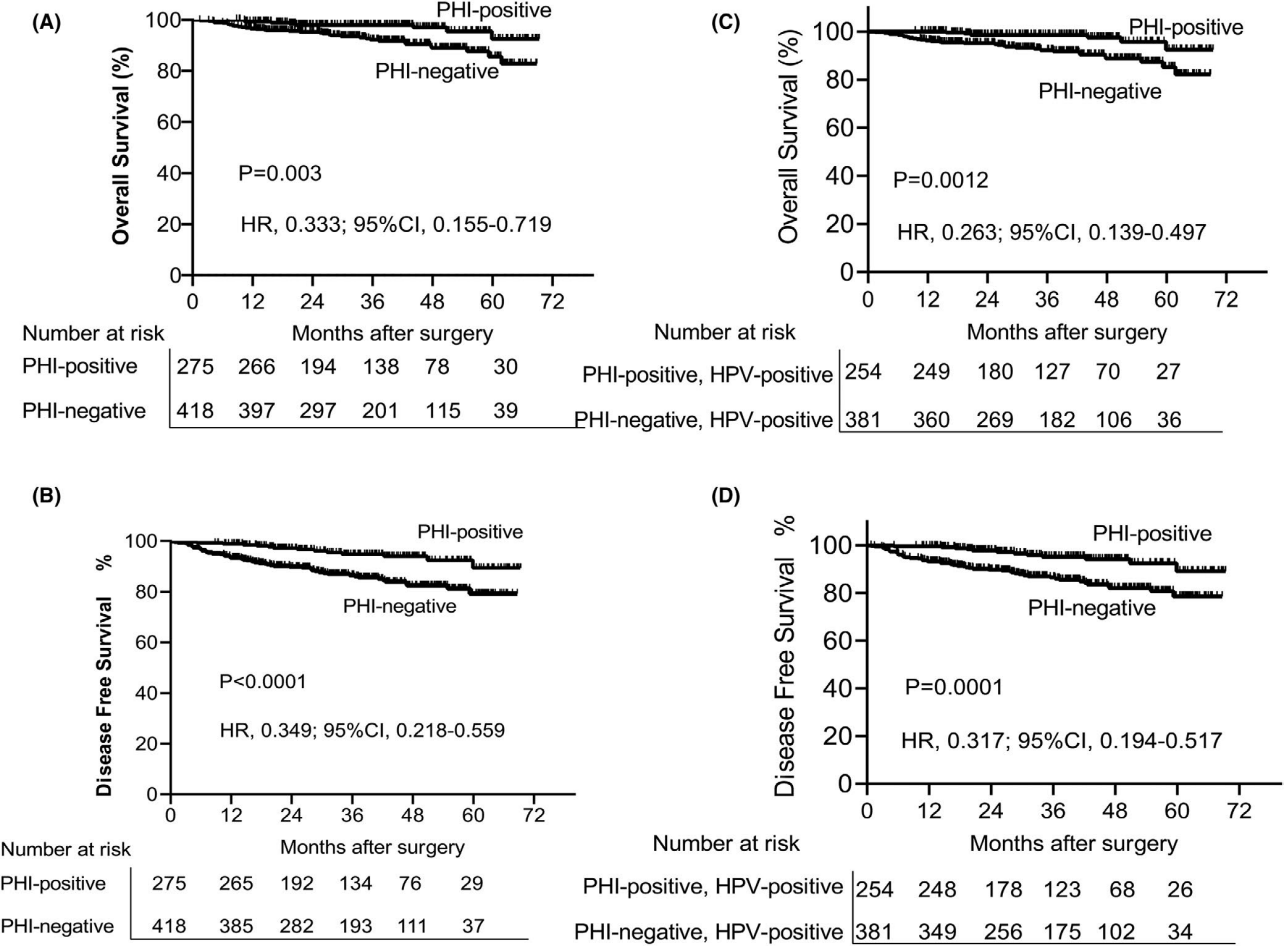
Prior hepatitis B virus infection Kaplan–Meier graph. (A) OS prognostic survival curve of patients with prior hepatitis B virus infection status. (B) DFS prognostic survival curve of patients with prior hepatitis B virus infection status. (C) OS prognostic survival curve of patients with prior hepatitis B virus infection and HPV infection status. (D) DFS prognostic survival curve of patients with prior hepatitis B virus infection and HPV infection status

**FIGURE 2:**
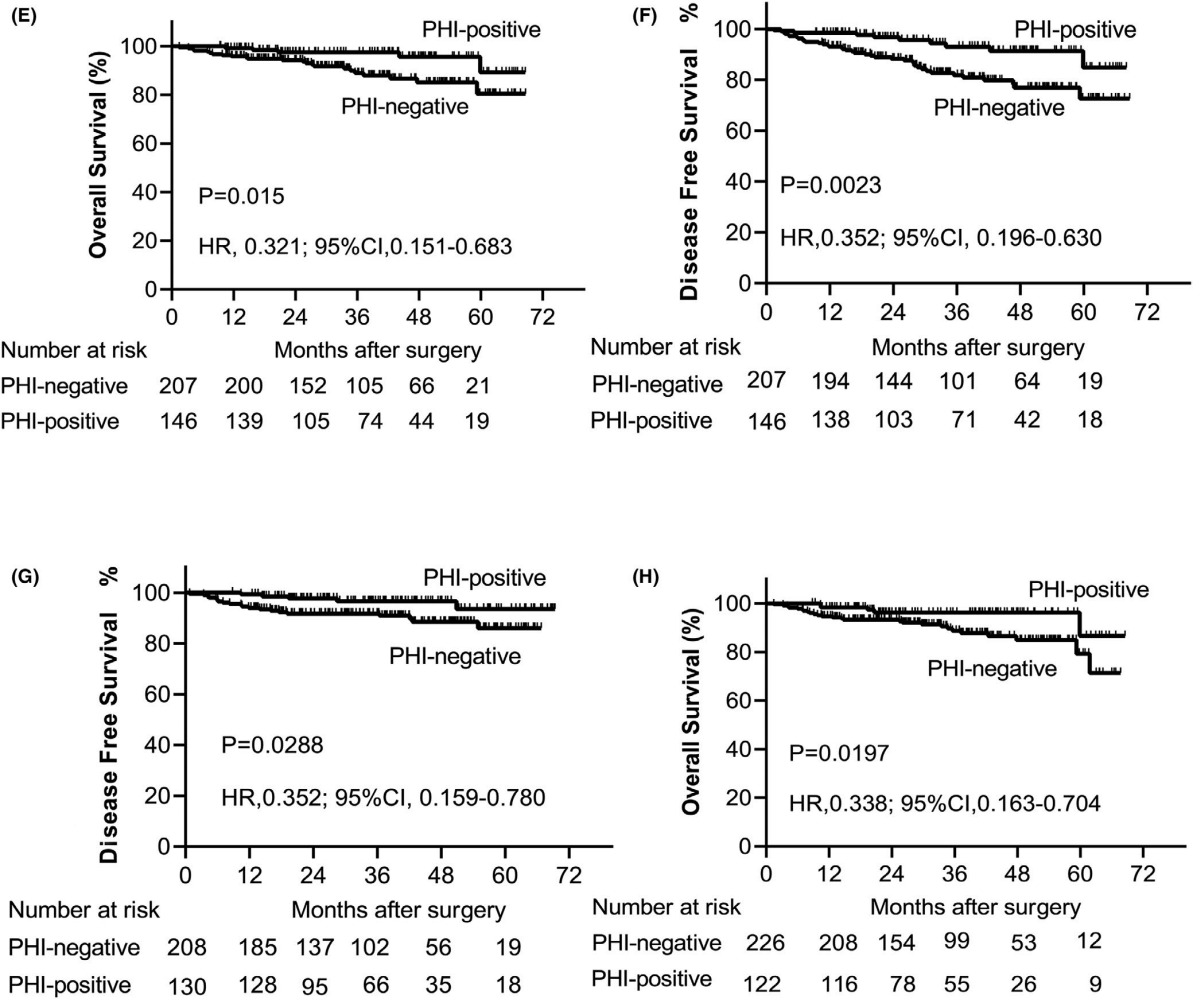
Prior hepatitis B virus infection status in subgroup analysis Kaplan–Meier graph. Kaplan–Meier curves show that Prior hepatitis B virus infection status was associated with favorable survival in patients with age and treatment in subgroup analysis. (E) Overall survival subdivided by prior hepatitis B virus infection in elder. (F) Disease‐free survival subdivided by prior hepatitis B virus infection in elder patients. (G) Overall survival subdivided by prior hepatitis B virus infection in surgery plus radiotherapy patients. (H) Disease‐free survival subdivided by prior hepatitis B virus infection in surgery plus radiotherapy patients

Patients were followed up for a median of 37.17 months (95% CI, 34.69–39.65). As shown in Figure 1A, the median OS of patients with PHI was 67.48 months, and that of patients without PHI was 63.55 months (*p* = 0.003). The same significant difference was also observed for DFS, with a median survival of 66.07 months for PHI‐positive and 60.10 months for PHI‐negative patients (*p* < 0.0001; Figure 1B). Considering HPV infection, the median OS was 67.76 months for HPV‐positive patients with PHI, 63.35 months for HPV‐positive patients without PHI. There was a trend toward greater survival benefit in patients positive for both HPV and PHI (*p* = 0.0012; Figure 1C). A similar trend was observed for DFS, with the most favorable survival outcomes manifested in HPV‐positive patients with PHI (median DFS 66.25 months), and HPV‐positive patients without PHI (median DFS 59.89 months). The difference in OS and DFS was statistically significant (*p* = 0.0001; Figure 1D). Subsequently, we considered the univariate survival analysis stratified by age and treatment. We found that in the elderly (Figure 2E,F), surgery plus radiotherapy (Figure 2G,H) patients with PHI status were associated with favorable OS and DFS. Young patients with PHI‐positive had a better DFS than those with PHI‐negative (*p* = 0.02, Table 4), but not for OS. However, there was no significant association between PHI infection in patients with surgery (*p* > 0.05, Table 4).

**TABLE 4:** Subgroup analysis by prior HBV infection status for overall survival and disease‐free survival in patients with cervical cancers

Variable	Overall survival			Disease‐free survival		
	Hazards Ratio	95% CI	*p*	Hazards Ratio	95% CI	*P*
Age						
≤47 years						
PHI‐negative		1			1	
PHI‐positive	0.321	0.151–0.683	**0.015**	0.352	0.196–0.630	**0.002**
>47 years						
PHI‐negative		1			1	
PHI‐positive	0.361	0.129–1.01	0.099	0.338	0.163–0.704	**0.020**
Treatment						
Surgery						
PHI‐negative		1			1	
PHI‐positive	0.411	0.132–1.282	0.168	0.765	0.324–1.809	0.550
Surgery plus radiotherapy						
PHI‐negative		1			1	
PHI‐positive	0.338	0.163–0.704	**0.020**	0.23	0.130–0.407	**<0.001**
HPV‐positive						
PHI‐negative		1			1	
PHI‐positive	0.263	0.139–0.497	**0.001**	0.317	0.194–0.517	**<0.001**

Note

PHI, prior hepatitis B virus (HBV) infection; 95% CI, 95% confidence interval bold values are statistically significant (*p* < 0.05).

## 4 DISCUSSION

Globally, cervical cancer (CC) is the second most common gynecological malignancy, next to uterine cancer and ovarian cancer.^14^ Infection with HPV has been the most well‐established cause contributing to the development of CC. Meanwhile, it is possible that other viral infections, chronic or acute, may also bear clinical relevance in the management of CC. One of the public health challenges China has been struggling against was HBV infection. In addition to hepatitis and liver cancer, HBV infection has been suggested as a prognostic factor in many extrahepatic malignancies.^15^, ^16^ For instance, HBV infection status was associated with poor DFS and OS in breast cancer.^12^ In this study, we analyzed the prognostic effect of HBV infection status in primary cervical cancer. Active HBV infection, indicated by HBsAg positivity, was observed in 12.7% of patients in our cohort. This incidence rate was consistent with the reported prevalence of HBsAg detection in 8%–15% of the population in this region,^17^ suggesting that primary CC is not associated with an increased rate of active HBV infection. We also found that HBV infection rate in CC patients was independent of other clinicopathological features including age, histology, FIGO stage, and HPV infection status. Next, through univariate *χ* ^2^ test and multivariate Cox regression analysis, we assessed the prognostic impact of the active or previous HBV infection with DFS and OS as endpoints. Interestingly, in contrast to reports of young breast cancer patients, in primary CC patients, HBsAg status appeared not to be associated with either survival outcome. It is possible, however, limitations in follow‐up length, cohort size, and relatively low HBsAg‐positive rate may have rendered this analysis underpowered to detect significant survival differences. Clinical validation is therefore necessary for this observation of the lack of prognostic relevance of active HBV infection. Survival analysis also showed that treatment with surgery plus radiotherapy was a poor prognostic factor in our cohort of CC patients. Despite advances in screening and treatment of early stage disease, a considerable proportion of CC patients is still diagnosed with advanced, recurrent, or persistent cervical cancer. For these patients, surgery in combination with radiotherapy is still the cornerstone of treatment. The age distribution was 45 (38–54) of patients in the surgery plus radiotherapy subgroup and 48 (41–55) for those treated only in surgery, with the latter being significantly lower (*χ* ^2^ *p* < 0.001). Also, 8.6% (30/350) of patients receiving surgery/radiotherapy had a tumor staged at ⅡB or higher, whereas those with >IIB CC comprised a significantly lower percentage (2.3%, 8/345) of patients who received surgery. Together, the worse prognosis observed in patients who underwent the combined regimen of surgery and radiotherapy could at least partially be attributed to more advanced age and disease stage.

Although HBsAg positivity appeared to be of no prognostic significance, we found an unexpected association between prior HBV infection (PHI) and reduced risk of death or recurrence. Multivariate survival analysis also showed that PHI was an independent predictor of OS and DFS (Table 3). To our knowledge, this study is the first to reveal a prognostic value of PHI in primary cervical cancer. It has been reported that patients with PHI manifested a lower incidence of postoperative death and extrahepatic metastasis than those without PHI.^18^ A previous meta‐analysis showed that in pancreatic cancer, the viral antigen/antibody pattern of PHI was not associated with higher risk.^19^ Some studies have also suggested that HBV vaccination is a positive predictor of cervical cancer,^20^ and most patients with previous hepatitis B infection have acquired immunity, which may contribute to a better prognosis. Other factors, such as genetic or epigenetic ones, may also affect the prognosis of patients with cervical cancer, although the association remains to be revealed. For example, recent studies have shown that overexpression of HBV interacting protein X (HBxIP) is associated with poorer clinical outcomes in patients with cervical cancer.^21^ HBxIP is the body's immune response induced after hepatitis B virus infection, HBV can modify the host gene through DNA integration near the insertion point, thereby causing the host cell genome to be unstable and produce an oncogenic fusion protein.^22^ HBx can promote cell proliferation, viability, and migration by regulating multiple signal pathways.^23^ However, in our research, prior HBV infections have already had a certain degree of immunity. The HBV level in the body may decrease, and the HBXIP in the body may also decrease. At the same time, studies have confirmed that hepatitis B patients can be effective in inhibiting virus replication by anti‐virus, and can prevent HBV complications, reduce the risk of liver cancer.^22^ In addition, HBV replication can promote residual immune cells and liver cells to produce tumor necrosis factor‐a (TNF‐a).^24^ Besides, several studies have investigated the prognostic role and clinical significance of programmed death‐1 (PDCD‐1) in treating cancer patients with PHI.^25^ In hepatocellular carcinoma patients, HBsAg‐specific B cells damaged during chronic HBV infection were functionally restored after HBsAg clearance by the administration of anti‐PD‐1. In high‐risk HPV‐associated cervical cancer, increased expression of PD‐1 and its ligand PD‐L1 is associated with dampened cell‐mediated immunity,^26^ suggesting that PD‐(L)1 has therapeutic potential in recurrent and/or metastatic CC patients not responding to immunotherapy. Therefore, it is possible that treatment with PD‐(L)1 inhibitors can also benefit CC patients with HBV infection. However, preclinical and clinical research is warranted to elucidate the underlying mechanisms and clinical validity of immunotherapy in HBV‐infected cervical cancer.

HPV infection is exceedingly prevalent in cervical cancer patients, with 99%–99.8% of patients testing positive for HPV. HPV has also been established as a major factor causing cervical cancer and precancerous lesions.^27^ Hepatitis B virus does not directly damage liver cells, but the immune response and immunopathological reaction triggered by it are the keys to the onset of hepatitis B.^28^ It has been shown that CD4+ cell count, CD4+/CD8+ ratio, and serum IL‐2 level were decreased in patients with chronic hepatitis B.^29^ When immunosuppression or immunodeficiency occurs, the incidence of genital HPV infection and HPV‐related diseases increases. Because HPV clearance depends mainly on cellular immune response, a damaged cellular immunity fosters immune escape of the tumor. Thus, we speculate that the interaction between HBV and HPV may have a certain effect on the progression of the disease. We analyzed the predictive value of PHI in HPV‐positive patients and found extended OS and DFS in the PHI‐positive, HPV‐positive subgroup when compared with PHI‐positive, HPV‐positive patients. In this study, we established the Kaplan–Meier models of DFS and OS for HBV with HPV infection status, but did not include 58 (8.3%) HPV‐negative patients with CC. The purpose of this study is to confirm the effect of PBI on prognosis and to clarify that the results of this study are not affected by HPV. Our study found that the prognosis of HPV complicated with PBI infection was better than that of PBI‐negative patients, which further confirmed that PBI infection is a favorable prognostic factor for cervical cancer.

There are some limitations in the study. First, our study was a retrospective study from a single‐institution experience, which may lead to selection bias. Second, the follow‐up time of this study may not be sufficiently long, as there were relatively few outcome events, which may have masked the effect of HBsAg on survival outcomes. The study was also limited by the relatively small cohort size and imbalance between subgroups due to the low incidence rate of HBsAg positivity and PHI. The small sample size renders this study more susceptible to inherent selection bias. Third, as a substantial lack of HBV‐DNA data, we did not analyze the effect of HBV‐DNA copy number on the survival of cervical cancer patients. Therefore, further prospective studies of a large sample of patients will be needed to confirm these results for clarity.

In conclusion, our cohort study provides the first clinical evidence suggesting prior HBV infection, but not detection of HBV surface antigen, as an independent favorable prognostic factor in patients with primary cervical cancer. Validation with larger cohorts and characterization of the underlying mechanisms await future investigations.

## CONFLICT OF INTEREST

The authors declare that they have no conflict of interest.

## AUTHOR CONTRIBUTIONS

Xiaoyan Feng: Conceptualization, methodology, software, data curation, and writing original draft. Huaiwu Lu: Co‐first author, conceptualization, data curation, project administration, and writing original draft preparation. Yuan Wei: Conceptualization, visualization, and investigation. Meimei Guan: Resources and supervision. Junyi Wang: Data curation and formal analysis. Changhao Liu: Resources and software. Tianran Shen: Conceptualization and methodology. Qingsong Chen: Conceptualization, validation, resources, and supervision. Qunxian Rao: Resources, conceptualization, methodology, and project administration.

## REVIEW BOARD/COMMITTEE APPROVAL

Institutional Review Board approval was obtained from the Medical Ethics Board at the Sun Yat‐sen Memorial Hospital.

## Data Availability

The data that supports the findings of this study are available in the supplementary material of this article.
